# Impact of traditional cooking methods on total phenolics, flavonoids, and antioxidant capacities of dark green leafy vegetables

**DOI:** 10.1371/journal.pone.0358478

**Published:** 2026-09-17

**Authors:** Bahar Yalçın Çeliktaş, Muazzez Garipağaoğlu, Şeyda Karaman Ersoy

**Affiliations:** 1 Fenerbahçe University, Faculty of Health Sciences, Department of Nutrition and Dietetics, Istanbul, Türkiye; 2 Haliç University, Institute of Graduate Education, Istanbul, Türkiye; 3 Fenerbahçe University, Faculty of Pharmacy, Department of Analytical Chemistry, Istanbul, Türkiye; University of Milan, ITALY

## Abstract

This study evaluated the effects of four domestic cooking methods—steaming, microwaving, low-water boiling and high-water boiling—on the phenolic composition and antioxidant capacities of five dark green leafy vegetables (sorrel, stinging nettle, spinach, Swiss chard and purslane). Total phenolics, phenolic acids, flavonoids and tannins were quantified spectrophotometrically, while antioxidant capacities were assessed by DPPH, ABTS and CUPRAC assays. Cooking induced species- and method-dependent changes in phenolic profiles. Total phenolic content significantly decreased in sorrel (p = 0.005), whereas other vegetables showed stable or slightly increased values. Phenolic acids increased markedly in stinging nettle and purslane, particularly after low-water boiling (+279.3% and +275.0%) and steaming (+153.2% and +159.5%). Flavonoid levels declined mainly under high-water boiling but remained stable in purslane. Tannin-associated phenolic compounds decreased in nettle and sorrel during boiling, whereas purslane exhibited increases across several treatments. Antioxidant capacities generally decreased with high-water boiling, while steaming and microwaving preserved or enhanced DPPH and CUPRAC activity in purslane. Strong correlations were observed between total phenolic content and antioxidant assays (r = 0.853–0.956). Overall, steaming and microwaving were identified as the most effective techniques for retaining phenolic compounds and antioxidant capacity, whereas high-water boiling caused the greatest losses. These findings highlight the importance of selecting appropriate cooking methods to preserve the nutritional quality of leafy vegetables.

## Introduction

Green leafy vegetables are widely recognized as “protective foods” due to their richness in vitamins, minerals, dietary fibre and phenolic compounds [1]. Their abundant bioactive constituents—including polyphenols, carotenoids and glucosinolates—have been associated with improved insulin sensitivity, reduced cardiovascular risk, antihypertensive effects and protection against several cancer types [2,3]. Current dietary guidelines recommend at least five servings (400 g/day) of fruits and vegetables, with two servings specifically derived from green leafy varieties [4].

Dark green leafy vegetables, in particular, exhibit strong biological activity owing to their diverse phytochemical profiles, including phenolic compounds, flavonoids, carotenoids, vitamins, minerals and essential fatty acids [5]. These profiles vary across species, contributing to different health-promoting properties. For instance, nettle

*(Urtica dioica)* contains high levels of phenolics and flavonoids- predominantly chlorogenic acid, caffeic acid derivatives, and rutin- that underpin its antioxidant activity [6], while spinach *(Spinacia oleracea)* provides lutein, β-carotene and phenolic acids — principally *p*-coumaric acid derivatives — alongside a unique flavonoid fraction dominated by patuletin and spinacetin glycosides-supporting immune and metabolic functions [7]. Swiss chard *(Beta vulgaris subsp. vulgaris)* demonstrates benefits for metabolic syndrome due to its betalain and phenolic content including syringic acid and vitexin-derived apigenin flavonoids- [8], and purslane *(Portulaca oleracea)*—rich in α-linolenic acid and vitamins E and C, caffeic acid, quercetin, kaempferol, and luteolin—exhibits anti-inflammatory and hypolipidemic effects [9]. Sorrel *(Rumex acetosa)* also shows high antioxidant potential attributable to its flavonoid and phenolic acid composition **—** particularly quercetin derivatives, chlorogenic acid, caffeic acid, and rutin **—** [10]. However, the levels of these bioactive compounds are highly influenced not only by species and genotype but also by cultivation conditions, post-harvest handling and particularly home cooking practices [11].

In everyday nutrition, most vegetables are consumed cooked, with boiling, steaming and microwaving being the most common household methods [12]. These thermal treatments can markedly influence the physical attributes, sensory quality and bioactive compounds of vegetables [13]. Depending on the species, raw material quality and applied method, heating may produce both beneficial and adverse outcomes [14]. Nutrient losses, tissue softening and colour changes often occur during cooking, yet bioavailability of certain bioactive components may increase and anti-nutritional factors may be reduced. Such changes are largely attributable to the disruption of the vegetable matrix, through which low molecular weight compounds released during heating can interact with nutrients, either reducing or enhancing their accessibility [15].

Although previous studies have examined the influence of individual cooking methods on total phenolic content and antioxidant activity, the findings remain inconsistent across species and experimental conditions [16–18]. Recent comprehensive reviews also highlight substantial variability in reported outcomes and emphasize the lack of a standardized methodology, making it difficult to identify the most beneficial cooking technique [19]. Moreover, limited research has simultaneously quantified key phenolic subclasses—phenolic acids, flavonoids and tannins—and linked them to multiple antioxidant assays within a unified experimental design. Evidence is particularly scarce for dark green leafy vegetables commonly consumed in Türkiye, such as nettle, spinach, sorrel, Swiss chard and purslane, especially under standardized domestic cooking conditions. To address these gaps, this study assessed the effects of four traditional cooking methods—steaming, microwaving, low-water boiling and high-water boiling—on total phenolic content, phenolic acids, flavonoids, tannins and antioxidant capacities (DPPH, ABTS, CUPRAC) in these vegetables. The three antioxidant assays were selected because they operate through distinct reaction mechanisms: DPPH and ABTS measure hydrogen atom transfer (HAT) and single electron transfer (SET)-based radical scavenging activity, while CUPRAC assesses electron transfer capacity via cupric ion reduction. Their combined use provides a more comprehensive and mechanistically diverse evaluation of antioxidant capacity than any single assay alone [20]. By integrating detailed phenolic characterisation with multiple antioxidant assays and correlation analysis, the study offers method- and species-specific insights that advance current understanding and inform practical dietary and culinary recommendations for optimising the nutritional quality of cooked leafy vegetables.

## Materials and methods

### Chemicals

All chemicals were of analytical grade and purchased from Merck (Darmstadt, Germany) and Sigma-Aldrich (St. Louis, USA). The reagents included Folin-Ciocalteu reagent, sodium carbonate (Na_2_CO_3_), sodium hydroxide (NaOH), sodium potassium tartrate (NaKC_4_H_4_O_6_), copper(II) sulfate pentahydrate (CuSO_4_·5H_2_O), gallic acid, ABTS [2,2′-azino-bis(3-ethylbenzothiazoline-6-sulfonic acid) diammonium salt], potassium persulfate (K_2_S_2_O_8_), DPPH [1,1-diphenyl-2-picrylhydrazyl], copper(II) chloride (CuCl_2_), neocuproine (Nc), ammonium acetate (NH_4_Ac), aluminum chloride (AlCl_3_), quercetin, sodium acetate (CH_3_COONa), sodium molybdate (Na_2_MoO4), sodium nitrite (NaNO2), hydrochloric acid (HCl) and Trolox [6-hydroxy-2,5,7,8-tetramethylchromane-2-carboxylic acid], and caffeic acid.

### Instruments

Analyses were performed using a UV–Vis spectrophotometer (PG Instruments T80 + , UK), incubator (Elektromag Lab., M6040 BP, Türkiye), analytical balance (Weightlab Instruments, WSA-224, Türkiye), centrifuge (Elektromag, M4812M, Türkiye), shaking water bath (Nükleon Laboratuvar, TT107–30 L, Türkiye), and vortex mixer (Dizgi Analitik, Türkiye). Micropipettes (10–100 µL and 100–1000 µL, Ertick, Türkiye) were used for precise liquid handling. Laboratory glassware (beakers, tubes, glass pipettes; ISOLAB, Germany) was employed in all assays. For sample preparation, a microwave oven (Arçelik MD 574, Türkiye), hand blender (Sinbo SHB 3107, Türkiye), and stainless-steel cooking pot (Karaca Galaxy Series, Türkiye) were utilized.

### Plant material and sample preparation

**Fig 1:**
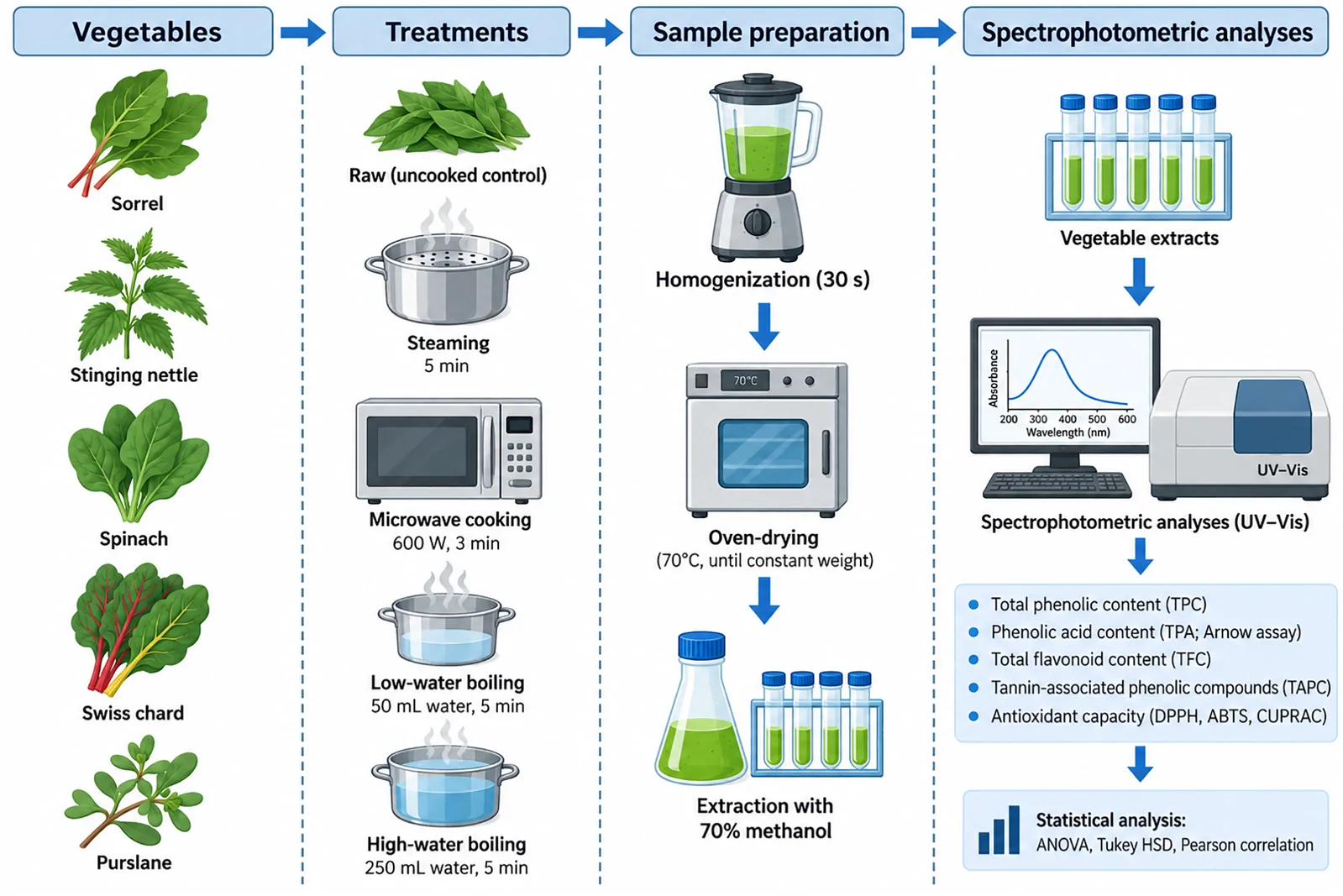
Graphical overview of the experimental study design.

Fresh samples of five dark green leafy vegetables-Nettle *(Urtica dioica),* Spinach *(Spinacia oleracea),* Sorrel *(Rumex acetosa),* Swiss chard *(Beta vulgaris subsp. cicla),* and Purslane *(Portulaca oleracea)*—were purchased from local farmers’ markets in Istanbul, Türkiye. Approximately 3 kg of each vegetable was procured. Immediately after purchase, all samples were cleaned, washed under running tap water, and gently dried with paper towels. To prevent nutrient loss, no storage was applied; samples were processed directly. Vegetables were chopped into uniform pieces and weighed into 100 g portions using an analytical balance to ensure consistency across experimental replicates. Each vegetable was divided into three independent sample portions, which were subjected separately to each cooking treatment, representing biological replicates. Subsequently, for each biological replicate, all chemical and antioxidant measurements were carried out in analytical triplicate to ensure measurement precision. The overall experimental workflow is illustrated in Fig 1.

### Cooking procedures

Cooking conditions were standardized according to previously published protocols with minor modifications [21–23]. The two boiling conditions were selected to reflect contrasting domestic practices commonly reported in the literature: low-water boiling (50 mL per 100 g) simulates minimal-water cooking as described by Turkmen et al. [21] and Şengül et al. [22], while high-water boiling (250 mL per 100 g) represents conventional excess-water boiling widely used in household settings [23]. These conditions were deliberately chosen to represent the full range of water-contact cooking, enabling assessment of the role of water volume as an independent variable in phenolic retention.

Low-water boiling: 100 g of vegetable samples were added to 50 mL of boiling distilled water in a stainless-steel pot, covered, and simmered for 5 min.

High-water boiling: 100 g of samples were cooked in 250 mL boiling distilled water for 5 min.

Steaming: 100 g of samples were placed in a metal strainer over 300 mL boiling distilled water, covered, and steamed for 5 min.

Microwave cooking: 100 g of vegetables with 15 mL distilled water were placed in a porcelain dish (25 cm diameter, 5 cm depth) and microwaved at 600 W for 3 min.

Cooking times of 3–5 min were selected to reflect typical household culinary practices reported in the literature for leafy vegetables [21,22], and to ensure visual and textural doneness without excessive heat exposure. These durations are consistent with times previously applied in comparative cooking studies on similar plant matrices [23]. It is acknowledged that optimal cooking time may vary among species; however, a standardised duration was applied across all vegetables to enable direct comparison of cooking method effects independent of time variables. After cooking, samples were cooled to room temperature, homogenized with a hand blender for 30 s and processed for further analysis.

### Dry matter determination

To eliminate the influence of variable moisture content among samples, all analytical data were expressed on a dry weight (DW) basis. Following each cooking treatment, homogenized samples were placed in pre-weighed aluminum dishes and dried in an incubator at 70 °C until a constant weight was achieved (approximately 48 h). Dry matter content was then calculated from the difference between the initial and final sample weights. Dry matter content was calculated using the following equation 1:


$$\begin{array}{c} \text{DM }(\%)\;=\;(\frac{{\text{W}}_{\text{2}}}{{\text{W}}_{\text{1}}})\;\times \text{ 100} \end{array}$$ (1)

where W_1_ is the fresh weight of the homogenized sample (g) and W_2_ is the weight of the sample after drying to constant mass (g).

The dried samples were subsequently used for extraction and further analyses; therefore, the results reflect the combined effects of cooking and subsequent drying.

### Preparation of extracts

The extraction procedure was adapted from Türkmen et al. [21] with minor modifications. Approximately 2 g of dried and homogenized raw or cooked vegetable samples were extracted with 10 mL of 70% methanol in a shaking water bath at 25 °C for 2 h. Extracts were centrifuged (5000 rpm, 15 min), and the supernatants were collected. The residues were re-extracted under the same conditions, and pooled supernatants were filtered through Whatman No. 1 filter paper. The clarified extracts were stored at −18 °C until analysis.

### Determination of total phenolic content

Total phenolic content (TPC) was determined using the Folin–Ciocalteu method described by Singleton et al. [24], implemented via a modified protocol incorporating copper-alkaline reagent solutions (Lowry A, B, and C). This modification is based on the principle originally described by Lowry et al. [25]. In this approach, an alkaline copper pre-treatment is applied prior to the addition of the Folin–Ciocalteu reagent. Although this modification may yield values that differ quantitatively from those obtained using the conventional Folin–Ciocalteu procedure [26], all samples and calibration standards were processed under identical analytical conditions, ensuring internal consistency for comparative purposes.

Three solutions were prepared: Lowry A solution: 2% (w/v) Na_2_CO_3_ dissolved in 0.1 M NaOH. Lowry B solution: 1% (w/v) NaKC_4_H_4_O_6_ and 0.5% (w/v) CuSO_4_. Lowry C solution: prepared by mixing 50 mL Lowry A with 1 mL Lowry B. Briefly, 0.1 mL of sample extract, 1.9 mL distilled water, and 2.5 mL Lowry C solution were added to test tubes and incubated at room temperature for 10 min. Subsequently, 0.25 mL of Folin-Ciocalteu reagent diluted 1:3 with distilled water was added. After 30 min incubation, absorbance was measured at 750 nm using the UV-Vis spectrophotometer against a blank. Gallic acid standards (6.00 × 10 ^−^ ^5^–3.00 × 10 ^−^ ^4^ mol/L) were subjected to the same analytical procedure, including the Lowry C pre-treatment, and were used to construct the calibration curve (y = 6100x + 0.0158, R^2^ = 0.9984). Results were calculated from the calibration curve and expressed as µmol gallic acid equivalents per gram dry weight (µmol GAE/g DW) according to Equation 2.


$$\begin{array}{c} \text{TPC }(\mu \text{mol}/\text{g GAE})=\;\frac{\text{Absorbance }\times \text{Dilution factor }\times \;\frac{\text{Total Volume }(\text{4}.\text{75 ml})}{\text{Sample volume }(\text{x ml})}\times \text{1000}}{\epsilon \text{ gallic acid }\left(\text{6100 }\frac{L}{mol\;cm}\;\right)\;\times \text{ m }(\text{g},\text{ dry mass of the vegetable})\;} \end{array}$$ (2)

### Determination of total flavonoid content

Total flavonoid content (TFC) was determined according to the spectrophotometric method developed by Chang et al. [27], with minor modifications. In brief, 0.5 mL of vegetable extract was mixed with 1.5 mL methanol, 0.1 mL of 10% aluminum chloride (AlCl_3_) solution, 0.1 mL of 1 M sodium acetate (CH_3_COONa), and 2.8 mL distilled water. The mixture was thoroughly vortexed and incubated at room temperature for 30 min. Following incubation, the absorbance was measured at 427 nm. Calibration was performed with quercetin standards (5.00 × 10 ^−^ ^6^-3.00 × 10 ^−^ ^5^ mol/L) (y = 21600x + 0.0056, R² = 0.998). Results were expressed as µmol QE/g DW (QE: Quercetin equivalent) with equation 3.


$$\begin{array}{c} \text{TFC }(\mu \text{mol}/\text{g QE})=\frac{\text{Absorbance }\times \text{Dilution factor }\times \;\frac{\text{Total Volume }(\text{5}\text{ mL})}{\text{Sample volume }(\text{x mL})}\times \text{1000}}{\epsilon \text{ quercetin }\left(\text{21600 }\frac{L}{mol\;cm}\right)\times \text{ m }(\text{g},\text{ dry mass of the vegetable})\;} \end{array}$$ (3)

### Determination of total phenolic acid content (Arnow Assay)

Total phenolic acids (TPA) were determined using the Arnow assay as described by Gawlik-Dziki et al. [28]. It should be noted that the Arnow assay selectively detects ortho-dihydroxy phenolic compounds — primarily caffeic acid, chlorogenic acid, and related catechol-type derivatives — through their reaction with molybdate-nitrite reagent under alkaline conditions. Phenolic acids lacking the ortho-dihydroxy (catechol) structural motif, such as gallic acid, ferulic acid, and p-coumaric acid, do not yield a chromogenic response in this assay. Therefore, the values reported here represent ortho-dihydroxy phenolic acid fractions rather than total phenolic acids in the broadest sense, and should be interpreted accordingly. Reaction mixtures contained 0.5 mL extract, 2.5 mL distilled water, 0.5 mL 0.5 M HCl, 0.5 mL Arnow reagent (10% (w/v) sodium molybdate and 10% (w/v) sodium nitrite), 0.5 mL 10% NaNO_2_, and 0.5 mL 1 M NaOH, adjusted to 5.0 mL with distilled water. Absorbance was measured at 490 nm. Caffeic acid standards (2.0 × 10 ^−^ ^5^-2.4 × 10 ^−^ ^4^ mol/L) were used for calibration (y = 3642x + 0.0451, R² = 0.9985). Results were expressed as µmol CAE/g DW (CAE: caffeic acid equivalent) by using the following equation 4.


$$\begin{array}{c} \text{TPA }(\mu \text{mol}/\text{g CAE})=\;\frac{\text{Absorbance }\times \text{Dilution factor }\times \;\frac{\text{Total Volume }(\text{5}\text{ mL})}{\text{Sample volume }(\text{x mL})}\times \text{1000}}{\epsilon \text{ caffeic acid }\left(\text{3642 }\frac{L}{mol\;cm}\right)\;\times \text{ m }(\text{g},\text{ dry mass of the vegetable})\;} \end{array}$$ (4)

### Determination of tannin-associated phenolic compounds

Effects on tannin-associated phenolic compounds (TAPC) were determined following Zargar et al. [29] using the Folin–Ciocalteu method. A 0.1 mL extract was diluted with 7.5 mL distilled water, then mixed with 0.5 mL Folin reagent and 1.0 mL 35% Na_2_CO_3_. After vortexing, dilution to 10 mL and incubation for 30 min, absorbance was measured at 725 nm. Gallic acid standards (5.00 × 10 ^−^ ^6^–3.00 × 10 ^−^ ^5^ mol/L) were used for calibration (y = 291497x – 0.0158, R^2^ = 0.9998). Results were expressed as µmol GAE/g DW according to equation 5. It should be noted that the Folin–Ciocalteu reagent reacts non-specifically with a broad range of reducing compounds, including non-tannin phenolics, ascorbic acid, reducing sugars, and amino acids [20]. In the absence of a prior tannin-specific isolation step – such as PVPP adsorption, protein precipitation, or condensed tannin-specific methods (e.g., vanillin–HCl or butanol–HCl assays) –the values reported here do not selectively quantify tannins; they are therefore designated as tannin-associated phenolic compounds (TAPC) and should be interpreted as operational Folin-reactive estimates within a comparative analytical framework. Furthermore, it is acknowledged that the Folin–Ciocalteu, AlCl_3_ (flavonoid), and Arnow (phenolic acid) assays employed in this study exhibit overlapping reactivity toward certain phenolic compound classes; consequently, the four parameters reported– TPC, TPA, TFC, and TAPC – represent operationally defined spectrophotometric fractions rather than strictly isolated biochemical categories. This limitation is inherent to colorimetric phenolic analysis and has been widely recognized in the literature [20]. Chromatographic approaches such as HPLC-DAD or LC-MS/MS would be required for definitive subgroup separation and are recommended for future studies.


$$\begin{array}{c} \text{TAPC }(\mu \text{mol}/\text{g GAE})=\;\frac{\text{Absorbance }\times \text{Dilution factor }\times \;\frac{\text{Total Volume }(\text{10 mL})}{\text{Sample volume }(\text{x mL})}\times \text{1000}}{\epsilon \text{ gallic acid }\left(\text{291497}\frac{L}{mol\;cm}\right)\times \text{ m }(\text{g},\text{ dry mass of the vegetable})\;} \end{array}$$ (5)

### ABTS radical scavenging assay

ABTS radical scavenging activity was evaluated according to Re et al. [30]. A 7 mM ABTS solution was prepared by dissolving 0.1920 g ABTS in distilled water and mixing with 0.0331 g K_2_S_2_O_8_ to obtain a 2.45 mM solution, kept in the dark for 12–16 h. The ABTS ⁺ · solution was diluted with methanol (1:10, v/v). For analysis, 2.9 mL methanol, 0.1 mL extract, and 1 mL ABTS ⁺ · solutions were mixed and incubated at room temperature for 6 min. Absorbance was recorded at 734 nm. Trolox standards (5.00 × 10 ^−^ ^6^-2.50 × 10 ^−^ ^5^ mol/L) were used for calibration (y = 26000x-0.0322, R² = 0.9965), and results were expressed as µmol Trolox equivalents per g dry weight (µmol TE/g DW) according to equation 6.


$$\begin{array}{c} {\text{TAC}}_{\text{ABTS}}\;(\mu \text{mol}/\text{g TE})=\;\frac{\text{Absorbance }\times \text{Dilution factor }\times \;\frac{\text{Total Volume }(\text{4}\text{ mL})}{\text{Sample volume }(\text{x mL})}\times \text{1000}}{\epsilon \text{ Trolox }\left(\text{26000}\frac{L}{mol\;cm}\right)\times \text{m }(\text{g},\text{ dry mass of the vegetable})\;} \end{array}$$ (6)

### DPPH radical scavenging assay

The DPPH assay was performed according to Çelik et al. [31]. A 0.5 mM methanolic DPPH solution was prepared by dissolving 11.71 mg DPPH in 100 mL methanol and stored in the dark. Reaction mixtures contained 0.5 mL DPPH solution, 0.15 mL extract, and 1.85 mL methanol. Samples were incubated at room temperature for 30 min, and absorbance was measured at 525 nm. Trolox standards (1.00 × 10 ^−^ ^5^–6.00 × 10 ^−^ ^5^ mol/L) were used for calibration (y = 12400x + 0.5828, R² = 0.9901). Results were expressed as µmol TE/g DW by using the following equation 7.


$$\begin{array}{c} {\text{TAC}}_{\text{DPPH}}\;(\mu \text{mol}/\text{g TE})=\;\frac{\text{Absorbance }\times \text{Dilution factor }\times \;\frac{\text{Total Volume }(\text{2}.\text{5}\text{ mL})}{\text{Sample volume }(\text{x mL})}\times \text{1000}}{\epsilon \text{ Trolox }\left(\text{12400 }\frac{L}{mol\;cm}\right)\times \text{ m }(\text{g},\text{ dry mass of the vegetable})\;} \end{array}$$ (7)

### CUPRAC assay

CUPRAC (cupric ion reducing antioxidant capacity) was measured following Apak et al. [32]. The assay mixture contained 1.0 mL CuCl_2_ (1.0 × 10 ^−^ ^2^ M), 1.0 mL Nc (7.5 × 10 ^−^ ^3^ M), and 1.0 mL NH_4_Ac buffer (1.0 M, pH 7.0), followed by 0.1 mL extract and 0.9 mL methanol. After vortexing and incubation for 30 min at room temperature, absorbance was measured at 450 nm. Trolox standards (1.00 × 10 ^−^ ^5^- 6.00 × 10 ^−^ ^5^ mol/L) were used for calibration (y = 15400x-0.0326, R² = 0.9970). Results were expressed as µmol TE/g DW according to equation 8.


$$\begin{array}{c} {\text{TAC}}_{\text{CUPRAC}}\;(\mu \text{mol}/\text{g TE})=\;\frac{\text{Absorbance }\times \text{Dilution factor }\times \;\frac{\text{Total Volume }(\text{4}.\text{0}\text{ mL})}{\text{Sample volume }(\text{x mL})}\times \text{1000}}{\epsilon \text{ Trolox }\left(\text{15400 }\frac{L}{mol\;cm}\right)\times \text{ m }(\text{g},\text{ dry mass of the vegetable})\;} \end{array}$$ (8)

For reference and comparison with literature values commonly expressed in mg/g, the µmol values reported in this study can be converted using the respective molecular weights of the reference standards: gallic acid (MW = 170.12 g/mol) for TPC and TAPC, caffeic acid (MW = 180.16 g/mol) for TPA, quercetin (MW = 302.24 g/mol) for TFC, and trolox (MW = 250.29 g/mol) for antioxidant capacity assays. For example, TPC values of 53.55 µmol GAE/g DW and 12.12 µmol GAE/g DW for raw sorrel and spinach correspond to approximately 9.11 mg GAE/g DW and 2.06 mg GAE/g DW, respectively.

The calibration curves and graphical summaries of cooking-induced changes are presented in Supplementary File 1.

### Statistical analysis

All statistical analyses were performed using IBM SPSS Statistics for Windows, Version 29.0 (IBM Corp., Armonk, NY, USA). Prior to analysis, the normality of the data distribution was assessed using skewness and kurtosis coefficients, and homogeneity of variances was evaluated using Levene’s test. One-way analysis of variance (ANOVA) was conducted to determine significant differences among groups. When a significant effect was observed, Tukey’s Honestly Significant Difference (HSD) post hoc test was applied for multiple comparisons. Relationships between continuous variables were examined using Pearson’s correlation analysis. A p-value < 0.05 was considered statistically significant for all analyses. The raw experimental data used for these statistical analyses are provided in S2 File.

## Results and discussion

### Dry matter content

The dry matter contents of raw and cooked samples are presented in Table 1. Among the uncooked vegetables, nettle showed the numerically highest dry matter content (9.56 ± 0.38%), while the remaining species ranged between 3.54% and 6.05%. These values are consistent with previously reported ranges of 7.2–14.6% for wild species [23] and 7.39–19.83% for cultivated leafy vegetables [33]. Variations in dry matter content were observed among cooking methods, with steaming and microwaving generally associated with higher values in some vegetables, while high-water boiling tended to yield lower values. The decreases observed during boiling are attributable to the leaching of water-soluble constituents into the cooking medium, including sugars, organic acids, soluble dietary fibre, soluble proteins, minerals, and low-molecular-weight phenolic compounds. Such losses can substantially reduce dry matter yield and may simultaneously alter the apparent concentration and proportional contribution of phenolic compounds in the extracts — independently of any direct effect of heat on phenolic stability. Species-specific variations in the magnitude of these losses likely reflect differences in cell wall architecture, tissue porosity, and the relative abundance of soluble solids.

**Table 1:** Dry matter (%) of raw and cooked vegetables.

Vegetables	Raw	Steaming	Microwaving	Low-water boiling	High-water boiling
Sorrel	5.25 ± 1.91	6.08 ± 0.84	6.99 ± 0.30	5.48 ± 0.17	4.63 ± 0.24
Stinging nettle	9.56 ± 0.38	12.68 ± 1.42	12.11 ± 1.22	11.08 ± 0.91	7.59 ± 0.49
Spinach	5.48 ± 0.94	4.96 ± 0.13	4.87 ± 0.65	4.41 ± 0.22	3.38 ± 0.40
Swiss chard	6.05 ± 0.79	6.01 ± 0.09	6.46 ± 0.37	4.75 ± 0.41	4.49 ± 0.15
Purslane	3.54 ± 0.58	3.84 ± 0.42	4.44 ± 0.75	4.43 ± 0.61	3.16 ± 0.43

*Dry matter values are expressed as mean ± standard deviation.*

### Effects on total phenolic content

The effects of different cooking methods on total phenolic content (TPC), phenolic acids, flavonoids and tannin-associated phenolic compounds are presented in Table 2. Among the raw samples, sorrel exhibited the highest TPC (53.55 ± 1.00 µmol GAE/g DW), followed by spinach, purslane, nettle and Swiss chard. Overall, cooking tended to reduce TPC; however, these changes were statistically significant only in sorrel (p = 0.005). No significant differences among cooking methods were observed for nettle, spinach, Swiss chard or purslane (p > 0.05).

**Table 2:** Effects of cooking methods on TPC, TPA, TFC, and TAPC of vegetables.

	Raw	Steaming	Microwaving	Low-water boiling	High-water boiling
**TPC (µmol GAE/g DW)**					
Sorrel	53.55 ± 1.00^a,A^	27.79 ± 11.56^ab,A^	23.45 ± 10.36^b,A^	20.55 ± 14.81^b,A^	13.88 ± 1.52^b,A^
Stinging nettle	9.58 ± 4.21^a,B^	8.20 ± 1.52^a,B^	5.69 ± 2.72^a,B^	3.33 ± 0.03^a,A^	5.89 ± 3.41^a,B^
Spinach	12.12 ± 5.64^a,B^	8.28 ± 1.95^a,B^	9.07 ± 0.91^a,B^	10.44 ± 1.50^a,A^	6.50 ± 1.79^a,B^
Swiss chard	8.34 ± 5.95^a,B^	6.06 ± 2.84^a,B^	5.53 ± 1.06^a,B^	5.89 ± 3.07^a,A^	5.84 ± 2.18^a,B^
Purslane	12.10 ± 8.54^a,B^	13.26 ± 1.36^a,AB^	11.16 ± 3.97^a,AB^	17.88 ± 13.67^a,A^	12.02 ± 3.45^a,AB^
**TPA (µmol CAE/g DW)**					
Sorrel	13.98 ± 8.17^a,A^	20.06 ± 5.48^a,A^	14.17 ± 2.82^a,A^	13.81 ± 3.89^a,A^	9.13 ± 1.92^a,A^
Stinging nettle	0.25 ± 0.22^a,B^	0.64 ± 0.39^a,B^	0.56 ± 0.24^a,B^	0.96 ± 0.45^a,B^	0.41 ± 0.06^a,B^
Spinach	3.11 ± 2.10^a,B^	2.43 ± 0.91^a,B^	2.45 ± 0.47^a,B^	3.56 ± 2.79^a,AB^	1.69 ± 0.27^a,B^
Swiss chard	2.20 ± 0.13^a,B^	1.79 ± 0.18^a,B^	2.10 ± 0.57^a,B^	2.04 ± 0.31^a,B^	1.34 ± 0.35^a,B^
Purslane	3.10 ± 1.17^a,B^	8.04 ± 1.67^a,AB^	5.47 ± 1.79^a,AB^	11.62 ± 9.35^a,A^	7.09 ± 1.53^a,AB^
**TFC (µmol QE/g DW)**					
Sorrel	4.88 ± 0.09^a,B^	4.73 ± 0.12^a,A^	4.53 ± 0.26^a,B^	4.68 ± 0.19^a,A^	4.62 ± 0.05^a,A^
Stinging nettle	2.05 ± 0.08^a,E^	2.00 ± 0.18^a,B^	2.02 ± 0.03^a,D^	1.86 ± 0.05^a,B^	1.80 ± 0.04^a,B^
Spinach	5.27 ± 0.10^a,A^	4.27 ± 0.36^b,A^	5.12 ± 0.08^a,A^	4.63 ± 0.19^ab,A^	4.30 ± 0.03^b,A^
Swiss chard	2.87 ± 0.11^a,D^	1.87 ± 0.20^b,B^	1.79 ± 0.24^b,D^	1.78 ± 0.10^b,B^	1.78 ± 0.16^b,B^
Purslane	4.05 ± 0.17^a,C^	4.08 ± 0.23^a,A^	3.98 ± 0.06^a,C^	4.19 ± 0.20^a,A^	4.08 ± 0.12^a,A^
**TAPC (µmol GAE/g DW)**					
Sorrel	1.87 ± 0.12^a,A^	1.65 ± 0.36^a,A^	1.50 ± 0.41^a,A^	1.61 ± 0.48^a,A^	1.00 ± 0.24^a,A^
Stinging nettle	0.19 ± 0.01^a,D^	0.10 ± 0.04^b,B^	0.02 ± 0.00^b,B^	0.03 ± 0.02^b,B^	0.02 ± 0.02^b,B^
Spinach	0.53 ± 0.07^a,C^	0.57 ± 0.43^a,B^	0.43 ± 0.03^a,B^	0.51 ± 0.19^a,B^	0.19 ± 0.11^a,B^
Swiss chard	0.86 ± 0.07^a,B^	0.45 ± 0.15^a,B^	0.64 ± 0.18^a,B^	0.56 ± 0.07^a,B^	0.56 ± 0.20^a,A^
Purslane	0.31 ± 0.01^a,D^	0.51 ± 0.17^a,B^	0.53 ± 0.12^a,B^	0.60 ± 0.14^a,B^	0.59 ± 0.20^a,A^

*Values are mean ± SD. TPC: total phenolic content; TPA: total phenolic acids; TFC: total flavonoid content; TAPC: tannin-associated phenolic compounds. TAPC values represent Folin-reactive reducing substances in the absence of PVPP precipitation and should not be interpreted as analytically isolated tannin fractions. Different lowercase letters indicate differences among cooking methods, and uppercase letters indicate differences among vegetables (ANOVA, p < 0.05).*

The greatest numerical reductions in TPC were observed following high-water boiling, ranging from 0.7% to 74.1% depending on the vegetable species. Microwaving also reduced TPC in several vegetables, particularly in sorrel (−56.2%) and nettle (−40.6%); however, except for sorrel, these changes were not statistically significant. Similar species-dependent responses to thermal processing have been reported previously, indicating that phenolic stability is strongly influenced by both plant characteristics and cooking conditions [34–37].

At the mechanistic level, the reductions in total phenolic content observed during cooking may be attributed to thermal degradation and oxidation of phenolic compounds induced by heat treatment [38]. Heat can also alter phenolic–matrix interactions by disrupting ester and glycosidic linkages that bind phenolic compounds to cell wall components, thereby increasing the release and extractability of bound phenolic fractions [39]. However, prolonged heating may promote degradation of phenolic compounds, resulting in lower measurable TPC values [38]. The differing responses among vegetable species suggest that the impact of cooking on TPC is influenced by the composition and distribution of phenolic compounds within the plant matrix. The comparatively greater stability observed in purslane relative to sorrel may therefore reflect species-specific differences in phenolic composition and tissue structure.

Beyond the nature of thermal degradation itself, the magnitude of phenolic loss is further modulated by the water volume used during cooking. The substantially greater water volume in high-water boiling (250 mL vs 50 mL per 100 g sample) creates a larger concentration gradient between the vegetable matrix and the surrounding medium, potentially intensifying leaching of water-soluble phenolics into the cooking liquid independent of heat exposure. Consequently, the greater losses observed under high-water boiling likely represent the combined effect of thermal degradation and solvent-mediated extraction rather than thermal effects alone. This is consistent with previous reports showing that water volume is a primary determinant of phenolic loss during boiling [40] and underscores the importance of water-to-vegetable ratio as an independent variable in cooking studies. It should also be noted that the cooking water was not collected or analysed in this study; therefore, the reductions observed under boiling conditions cannot be attributed solely to thermal degradation, as migration of water-soluble phenolics into the cooking medium may have contributed to the apparent losses.

### Effects on phenolic acids

Phenolic acid content followed a similarly species-dependent response. Sorrel contained the highest levels in the raw state (13.98 ± 8.17 µmol CAE/g DM), followed by spinach, purslane, Swiss chard and nettle. Although steaming resulted in a numerical increase in phenolic acids in sorrel (+43.5%) and high-water boiling caused a numerical decrease (−34.7%), neither change was statistically significant (p > 0.05). Similarly, the apparent decreases in Swiss chard and the large numerical increases in nettle and purslane under low-water boiling (+279.3% and +275.0%, respectively) and steaming (+153.2% and +159.5%) did not reach statistical significance (p > 0.05), likely due to the high variability within groups.

The numerical increases observed in some vegetables may be associated with heat-induced release of previously bound phenolic compounds [41]. Thermal processing can alter phenolic–matrix interactions by disrupting ester and glycosidic linkages that bind phenolic compounds to cell wall components, thereby increasing the extractability of phenolic acids [42]. Conversely, reductions observed under boiling conditions may reflect the migration of water-soluble phenolic compounds into the cooking medium, particularly when larger volumes of water are used [38]. Similar shifts between bound and soluble phenolic fractions following thermal processing have been reported in other plant-based foods, including legumes and leafy vegetables [41,43]. Because the cooking water was not collected or analysed in the present study, the relative contributions of enhanced extractability, thermal degradation and leaching could not be distinguished. Overall, steaming and low-water boiling tended to better preserve phenolic acids than high-water boiling, although these differences were not statistically significant.

### Effects on total flavonoids

Flavonoid content also varied according to vegetable type and cooking method. Raw spinach exhibited the highest levels (5.27 ± 0.10 µmol QE/g DW), followed by sorrel, purslane, Swiss chard and nettle (p < 0.001). Swiss chard experienced significant reductions across all cooking methods (34–38%; p < 0.001), while purslane largely retained its flavonoids, with small numerical increases under some conditions. In spinach, steaming and high-water boiling resulted in significant reductions of 18–19% compared with raw samples (p < 0.05), whereas microwaving produced values that were not significantly different from the raw state. Nettle also exhibited reductions across all cooking methods, with the greatest loss observed following high-water boiling (p = 0.034).

These findings are consistent with previous studies showing that flavonoid retention during cooking is influenced by both processing conditions and the characteristics of the plant matrix [13,38,42,44,45]. Thermal treatment may affect the stability and extractability of flavonoid compounds through structural modifications of plant tissues and changes in flavonoid–matrix interactions [38]. Differences in the magnitude and direction of flavonoid changes among vegetable species therefore likely reflect variations in tissue structure and flavonoid composition. Overall, steaming appeared to preserve flavonoids more effectively than boiling, whereas the effects of microwaving varied among vegetable species [40].

### Effects on tannin-associated phenolic compounds

As noted in the Methods section, the values reported for tannin-associated phenolic compounds represent Folin-reactive reducing substances rather than analytically isolated tannin fractions and should therefore be interpreted within a comparative framework. Among the raw samples, sorrel exhibited the highest levels (1.87 µmol GAE/g DW), followed by Swiss chard, spinach, purslane and nettle.

Numerically, high-water boiling was associated with the greatest reductions in tannin-associated phenolic compounds, resulting in decreases of 46% in sorrel, 64% in spinach, 89% in nettle and 35% in Swiss chard. However, these changes were statistically significant only in nettle (p < 0.05). Sorrel largely maintained its tannin-associated phenolic content following steaming and microwaving, whereas purslane showed numerical increases under all cooking methods, although these changes did not reach statistical significance (p > 0.05).

Previous studies have similarly reported substantial reductions in tannin content following boiling and other thermal treatments [46–49]. The observed decreases may be associated with thermal degradation and the migration of water-soluble Folin-reactive compounds into the cooking medium, whereas apparent increases may reflect enhanced extractability of previously bound phenolic constituents during heat treatment. Because no tannin-specific isolation step was applied and cooking water was not analysed, the relative contributions of degradation, leaching and changes in extractability could not be distinguished in the present study.

### Changes in antioxidant capacities (DPPH, ABTS, CUPRAC)

The effects of different cooking methods on DPPH, ABTS and CUPRAC antioxidant capacities are presented in Table 3. In raw form, sorrel exhibited the highest DPPH activity (58.47 µmol TE/g DW), whereas the remaining vegetables ranged from 1.55 to 31.56 µmol TE/g DW, consistent with previously reported values of 2.3–77.2 µmol TE/g DW in leafy vegetables [50]. Cooking produced species-dependent responses. In purslane, steaming produced a statistically significant increase compared with raw samples (+60.2%; p < 0.05), while the increases observed with microwaving (+14.5%) and low-water boiling (+81.0%) did not reach statistical significance (p > 0.05). In sorrel, Swiss chard and nettle, the observed reductions across cooking methods were not statistically significant (p > 0.05). In spinach, high-water boiling resulted in a significant decrease compared with raw (p < 0.05), while other methods did not differ significantly. Such variability aligns with earlier findings indicating that thermal processes may either enhance or reduce DPPH activity depending on vegetable matrix and heat exposure [22,51–53]. For example, substantial increases (99–111%) have been reported after boiling tropical greens [51], while prolonged boiling has been shown to either improve or drastically reduce antioxidant capacity depending on the species [22]. Meta-analytic evidence similarly suggests that steaming generally increases DPPH activity, whereas boiling and microwaving often lead to declines [53,54]. Overall, these results demonstrate that thermal processing can both enhance and diminish DPPH capacity, largely due to structural and compositional changes affecting the release and stability of antioxidant compounds.

**Table 3:** Effects of different cooking methods on the antioxidant capacities of vegetables.

	Raw	Steaming	Microwaving	Low-water boiling	High-water boiling
**DPPH (µmol TE/g DW)**					
Sorrel	58.47 ± 3.48^a,A^	45.11 ± 17.60^a,A^	42.88 ± 17.43^a,A^	39.26 ± 28.09^a,AB^	24.44 ± 2.71^a,B^
Stinging nettle	1.55 ± 0.03^a,C^	1.12 ± 0.29^a,B^	1.01 ± 0.26^a,B^	1.19 ± 0.34^a,B^	1.35 ± 0.35^a,C^
Spinach	5.06 ± 0.27^a,C^	3.86 ± 0.85^ab,B^	4.72 ± 0.14^ab,B^	4.87 ± 0.02^ab,B^	3.63 ± 0.93^b,C^
Swiss chard	4.12 ± 1.64^a,C^	2.93 ± 1.49^a,B^	2.67 ± 0.84^a,B^	4.22 ± 0.30^a,B^	3.13 ± 0.89^a,C^
Purslane	31.56 ± 1.94^b,B^	50.54 ± 1.35^ab,A^	36.14 ± 9.80^ab,A^	57.12 ± 15.19^a,A^	43.87 ± 5.22^ab,A^
**ABTS (µmol TE/g DW)**					
Sorrel	55.79 ± 3.28^a,A^	33.89 ± 9.68^a,A^	29.48 ± 14.83^a,A^	34.45 ± 31.43^a,A^	18.03 ± 6.83^a,A^
Stinging nettle	5.03 ± 0.27^a,C^	4.12 ± 0.92^ab,B^	1.96 ± 0.33^b,B^	1.80 ± 1.50^b,A^	2.18 ± 0.34^b,B^
Spinach	10.72 ± 0.14^a,C^	6.07 ± 2.03^b,B^	8.91 ± 0.62^ab,AB^	7.86 ± 1.18^ab,A^	5.66 ± 1.71^b,B^
Swiss chard	7.37 ± 0.36^a,C^	2.38 ± 1.94^b,B^	2.37 ± 1.24^b,B^	3.05 ± 1.29^b,A^	1.69 ± 1.10^b,B^
Purslane	32.45 ± 1.04^a,B^	30.91 ± 11.04^a,A^	26.17 ± 6.56^a,A^	24.02 ± 11.17^a,A^	17.49 ± 1.19^a,A^
**CUPRAC (µmol TE/g DW)**					
Sorrel	106.61 ± 0.91^a,A^	86.18 ± 27.97^a,A^	71.15 ± 28.92^a,A^	59.95 ± 42.36^a,A^	43.48 ± 1.77^a,A^
Stinging nettle	1.21 ± 0.06^a,C^	0.93 ± 0.23^ab,B^	0.46 ± 0.06^b,B^	0.58 ± 0.04^b,B^	0.59 ± 0.25^b,B^
Spinach	10.84 ± 0.08^a,C^	6.02 ± 1.61^b,B^	8.30 ± 0.56^ab,B^	7.06 ± 1.04^b,AB^	4.89 ± 1.41^b,B^
Swiss chard	9.13 ± 1.31^a,C^	4.06 ± 2.51^b,B^	3.92 ± 1.57^b,B^	5.53 ± 1.13^ab,B^	4.53 ± 1.42^b,B^
Purslane	62.44 ± 1.80^a,B^	62.94 ± 15.66^a,A^	35.34 ± 10.07^a,AB^	79.00 ± 61.77^a,A^	42.32 ± 8.02^a,A^

*Values are presented as mean ± SD. Different lowercase letters indicate significant differences among cooking methods, while uppercase letters indicate differences among vegetables within each method (ANOVA, p < 0.05).*

For ABTS, sorrel again showed the highest antioxidant capacity in the raw state (55.79 µmol TE/g DW), with other vegetables ranging between 5.03 and 32.45 µmol TE/g DW—like previous values reported for wild edible plants [54,55]. Cooking generally reduced ABTS capacity, with significant decreases observed in nettle, spinach and Swiss chard (p < 0.01). High-water boiling was associated with the greatest numerical losses (46–77%); however, statistically significant reductions were observed only in nettle, spinach and Swiss chard (p < 0.05). In sorrel and purslane, no significant differences were detected among cooking methods (p > 0.05). Steaming and microwaving resulted in comparatively lower declines and helped preserve activity in purslane. Prior studies similarly reported 16–25% losses in steamed and microwaved broccoli [45], 10–30% reductions in boiled spinach and zucchini [55], and mixed effects—including both decreases and modest increases—depending on cooking method and vegetable type [56]. Collectively, these findings indicate that ABTS responses vary with species and processing conditions, with high water-contact methods intensifying losses, while microwaving and occasionally steaming better preserve antioxidant capacity.

CUPRAC values showed comparable trends. Sorrel exhibited the highest activity in raw form (106.61 ± 0.91 µmol TE/g DW), followed by purslane, spinach, Swiss chard and nettle. Cooking reduced CUPRAC capacity in nettle, spinach and Swiss chard, with statistically significant decreases observed across most methods (p < 0.05). In sorrel and purslane, no statistically significant differences were detected among cooking methods (p > 0.05), despite large numerical variations. The greatest numerical losses were observed under high-water boiling (−59.2% in sorrel; −54.9% in spinach), and under microwaving in nettle (−62%) and Swiss chard (−57%), the latter two being statistically significant. These trends are consistent with earlier studies showing that CUPRAC responses vary widely depending on vegetable type, heating time and cellular structure [18, 57, 58]. Reported increases of several hundred percent after boiling or microwaving artichoke [57] contrast with large losses in onions under similar treatments [58]. Increases—such as those observed in purslane—likely result from heat-induced disruption of cell walls and subsequent release of bound phenolic compounds that enhance measurable antioxidant capacity.

The relationships between phenolic compounds and antioxidant capacities under different cooking methods are shown in Table 4. In raw vegetables, total phenolic content exhibited very strong correlations with all antioxidant assays (DPPH, ABTS and CUPRAC; r = 0.911–0.920). Phenolic acids also showed similarly strong associations (r = 0.883–0.902), while tannin-associated phenolic compounds were significantly correlated with both phenolic compounds and antioxidant capacity. However, given that both tannin-associated phenolic compounds and TPC were estimated using Folin–Ciocalteu chemistry, this relationship should be interpreted with caution, as it may partly reflect shared analytical reactivity rather than independent biological associations. Flavonoids, in contrast, showed only moderate correlations.

**Table 4:** Correlations between phenolic compounds and antioxidant capacity according to cooking methods.

		TPC	TPA	DPPH	ABTS	CUPRAC	TFC	TAPC
Raw	1- TPC	1						
	2- TPA	.816**	1					
	3- DPPH	.912**	.897**	1				
	4- ABTS	.911**	.902**	.991**	1			
	5- CUPRAC	.920**	.883**	.996**	.996**	1		
	6- TFC	0.477	.654**	.520**	.555*	.533*	1	
	7- TAPC	.718**	.902**	.740**	.743**	.728**	0.447	1
Steaming	1- TPC	1						
	2- TPA	.955**	1					
	3- DPPH	.767**	.816**	1				
	4- ABTS	.822**	.869**	.953**	1			
	5- CUPRAC	.883**	.933**	.948**	.978**	1		
	6- TFC	.623*	.689**	.669**	.717**	.706**	1	
	7- TAPC	.877**	.915**	.613*	.696**	.783**	.652**	1
Microwaving	1- TPC	1						
	2- TPA	.931**	1					
	3- DPPH	.860**	.882**	1				
	4- ABTS	.871**	.851**	.983**	1			
	5- CUPRAC	.956**	.971**	.951**	.937**	1		
	6- TFC	0.483	0.492	0.466	.552*	0.48	1	
	7- TAPC	.876**	.934**	.757**	.735**	.887**	0.396	1
Low-water boiling	1- TPC	1						
	2- TPA	.876**	1					
	3- DPPH	.845**	.833**	1				
	4- ABTS	.935**	.822**	.856**	1			
	5- CUPRAC	.926**	.925**	.937**	.853**	1		
	6- TFC	.617*	.623*	.562*	.584*	.522*	1	
	7- TAPC	.648**	.677**	.559*	.776**	.516*	.583*	1
High-water boiling	1- TPC	1						
	2- TPA	.826**	1					
	3- DPPH	.757**	.805**	1				
	4- ABTS	.854**	.867**	.861**	1			
	5- CUPRAC	.873**	.953**	.917**	.913**	1		
	6- TFC	.651**	.717**	.591*	.754**	.696**	1	
	7- TAPC	.580*	.839**	.522*	.586*	.724**	0.446	1

Pearson Correlation Test, *p < 0.05, **p < 0.001, TPC: Total phenolic content; TPA: Total phenolic acids; TFC: Total flavonoids; TAPC: Tannin-associated phenolic compounds (estimated by Folin–Ciocalteu colorimetry).

Similar patterns were observed under steaming and microwaving, whereas correlations weakened under high-water boiling, possibly due to reduced extractability or loss of compounds. The dominant antioxidant mechanism in raw and mildly processed samples — reflected by the strong correlations between TPC and DPPH, ABTS, and CUPRAC (r = 0.911–0.920) — is consistent with hydrogen atom transfer (HAT) and single electron transfer (SET) pathways mediated by phenolic hydroxyl groups. However, the partial weakening of these correlations under high-water boiling conditions may indicate an increasing contribution of thermally-derived antioxidant compounds, including Maillard reaction products such as reductones and melanoidins, which are known to exhibit DPPH and ABTS radical scavenging activity independently of native phenolic content [12]. Such thermally-generated antioxidants may partly compensate for phenolic losses in boiled samples, obscuring a direct structure-activity relationship between TPC and measured antioxidant capacity. Overall, these findings suggest that phenolic compounds are major contributors to antioxidant capacity under the present conditions; however, these associations should be interpreted with caution, as other compounds and assay-specific responses may also influence the results. These observations are consistent with previous studies reporting strong associations between phenolic content and antioxidant activity in leafy vegetables [50, 59, 60].

This study has several limitations that should be considered when interpreting the findings. First, plant samples were obtained from a single source and did not account for potential seasonal or batch-related variability, which may limit the generalizability of the results. Second, samples were subjected to oven drying prior to extraction; therefore, the observed changes reflect the combined effects of cooking and subsequent drying, rather than cooking alone. In addition, the use of spectrophotometric assays provided overall estimates of phenolic content and antioxidant capacity, but did not allow for compound-specific identification. Finally, although biological and analytical replicates were applied, the relatively high variability observed in some parameters may reflect inherent heterogeneity in plant matrices. The spectrophotometric assay used for tannin-associated phenolic compound determination was based on Folin–Ciocalteu chemistry without prior tannin-specific precipitation (e.g., PVPP treatment) and therefore lacked selectivity for true tannin fractions. More broadly, the Folin–Ciocalteu, AlCl_3_ and Arnow methods used for TPC, TFC and TPA determination are known to exhibit overlapping reactivity toward shared phenolic substrates. Consequently, the reported parameters should be interpreted as operationally defined spectrophotometric fractions rather than biochemically distinct compound classes [20]. Additionally, cooking water was not retained or analysed. Therefore, the reductions observed under boiling conditions may partly reflect migration of soluble phenolic compounds into the cooking medium rather than true chemical degradation. In culinary applications where the cooking liquid is consumed, such as soups and stews, these apparent losses may represent redistribution rather than elimination of bioactive compounds.

## Conclusion

This study demonstrated that dark green leafy vegetables are important sources of phenolic compounds, flavonoids, tannin-associated phenolic compounds and antioxidant activity, and that their retention is influenced by the cooking method applied. Among the evaluated techniques, steaming and microwaving generally resulted in better preservation of phenolic compounds and antioxidant capacity, whereas high-water boiling was associated with greater reductions in most bioactive parameters. In some vegetables, low-water boiling was associated with higher measured phenolic acid values, suggesting that processing conditions may influence the availability of specific phenolic constituents. Overall, cooking methods involving limited water contact and shorter heating times appeared more favourable for preserving the bioactive properties of leafy vegetables. Together with evidence from previous studies indicating improved retention of sensory attributes under these conditions, the present findings support steaming and microwaving as favourable cooking methods for dark green leafy vegetables. However, the observed changes reflect the combined effects of cooking and the subsequent drying step applied prior to analysis. In addition, cooking water was not collected or analysed; therefore, apparent reductions observed under boiling conditions may partly reflect migration of soluble compounds into the cooking medium rather than true degradation. Despite these limitations, the findings provide practical insights for both household food preparation and institutional catering settings, where the preservation of bioactive compounds is an important consideration. Future studies incorporating different cooking durations, alternative processing techniques, cooking-water analysis and compound-specific analytical approaches are warranted to further elucidate the mechanisms underlying these changes and to support evidence-based dietary recommendations.

## Supporting information

S1 File Calibration curves for all spectrophotometric assays and graphical summaries of cooking-induced changes in phenolic compounds and antioxidant capacities. (DOCX)

S2 File Raw experimental data used for statistical analyses, including all replicate measurements for phenolic compounds and antioxidant capacity assays. (XLSX)
