# Supplementary material for: Impact of traditional cooking methods on total phenolics, flavonoids, and antioxidant capacities of dark green leafy vegetables
Source: PLoS One. 2026 Sep 17;21(9):e0358478. doi: 10.1371/journal.pone.0358478 (PMC13585253; doi:10.1371/journal.pone.0358478)
Supplement: Supplementary file 1 [file pone.0358478.s001.docx]

**Impact of Traditional Cooking Methods on Total Phenolics, Flavonoids, and Antioxidant Capacities of Dark Green Leafy Vegetables**

**Supplementary File I**

**CALIBRATION CURVES**

Gallic acid

Absorbance (750 nm)

| Concentration (mol/L) | Absorbans |
| --- | --- |
| 6e-05 | 0.365 |
| 0.00012 | 0.784 |
| 0.00018 | 1.102 |
| 0.00024 | 1.462 |
| 0.0003 | 1.856 |

**Figure S1.** Calibration curve of gallic acid for total phenolic content (TPC) determination using the Folin–Ciocalteu method.

Quercetin

| Concentration (mol/L) | Absorbance (427 nm) |
| --- | --- |
| 5e-06 | 0.1136 |
| 1e-05 | 0.2316 |
| 1.5e-05 | 0.3696 |
| 2e-05 | 0.4376 |
| 2.5e-05 | 0.5456 |
| 3e-05 | 0.6536 |

**Figure S2.** Calibration curve of quercetin prepared by the flavonoid determination method

Caffeic acid

| Concentration (mol/L) | Absorbance (nm) |
| --- | --- |
| 2e-05 | 0.1 |
| 4e-05 | 0.207 |
| 6e-05 | 0.267 |
| 8e-05 | 0.337 |
| 0.00024 | 0.917 |

**Figure S3.** Calibration curve of caffeic acid obtained by the Arnow assay.

Gallic acid

| Concentration (mol/L) | Absorbance (725 nm) |
| --- | --- |
| 5.88e-07 | 0.156 |
| 1.176e-06 | 0.331 |
| 1.764e-06 | 0.493 |
| 2.352e-06 | 0.667 |
| 2.94e-06 | 0.845 |

**Figure S4.** Calibration curve of gallic acid obtained by the TAPC method.

Trolox

| Concentration (mol/L) | Absorbance (734 nm) |
| --- | --- |
| 5e-06 | 0.0978 |
| 1e-05 | 0.2378 |
| 1.5e-05 | 0.3378 |
| 2e-05 | 0.4978 |
| 2.5e-05 | 0.6178 |

**Figure S5.** Calibration curve of Trolox for the ABTS assay.

Trolox

| Concentration (mol/L) | Absorbance ( 525 nm) |
| --- | --- |
| 5e-06 | 0.6448 |
| 1.5e-05 | 0.7388 |
| 2.5e-05 | 0.8928 |
| 3.5e-05 | 1.0168 |
| 4.5e-05 | 1.1508 |
| 5.5e-05 | 1.2648 |
| 6e-05 | 1.3268 |

**Figure S6.** Calibration curve of Trolox prepared by the DPPH method.

Trolox

| Concentration (mol/L) | Absorbance (450 nm) |
| --- | --- |
| 1e-05 | 0.1354 |
| 2e-05 | 0.2927 |
| 3e-05 | 0.41 |
| 4e-05 | 0.6073 |
| 5e-05 | 0.7646 |
| 6e-05 | 0.9219 |

**Figure S7.** Calibration curve of Trolox prepared by the CUPRAC method.

**Graphical Presentation of Phenolic Compounds and Antioxidant Capacity Results**

Total phenolic content (µmol GAE/g DW)

|  | Raw | Steaming | Microwave cooking | Low-water boiling | High-water boiling |
| --- | --- | --- | --- | --- | --- |
| Sorrel | 53.55 | 27.79 | 23.45 | 20.55 | 13.88 |
| Nettle | 9.58 | 8.2 | 5.56 | 3.33 | 5.89 |
| Spinach | 12.12 | 8.28 | 9.07 | 10.44 | 6.5 |
| Swiss chard | 8.34 | 6.06 | 5.53 | 5.89 | 5.84 |
| Purslane | 12.1 | 13.26 | 11.16 | 17.88 | 12.02 |

Figure S8. Total phenolic contents of vegetables in raw and cooked forms.

Figure S9. Total phenolic acid contents of vegetables in raw and cooked forms

Total phenolic acid content (µmol CAE/g DW)

|  | Raw | Steaming | Microwave cooking | Low-water boiling | High-water boiling |
| --- | --- | --- | --- | --- | --- |
| Sorrel | 13.98 | 20.06 | 14.17 | 13.81 | 9.13 |
| Nettle | 0.25 | 0.64 | 0.56 | 0.96 | 0.41 |
| Spinach | 3.11 | 2.43 | 2.45 | 3.56 | 1.69 |
| Swiss chard | 2.2 | 1.79 | 2.1 | 2.04 | 1.34 |
| Purslane | 3.1 | 8.04 | 5.47 | 11.62 | 7.09 |

Total flavonoid content (µmol QE/g DW)

|  | Raw | Steaming | Microwave cooking | Low-water boiling | High-water boiling |
| --- | --- | --- | --- | --- | --- |
| Sorrel | 4.88 | 4.73 | 4.53 | 4.68 | 4.62 |
| Nettle | 2.05 | 2 | 2.02 | 1.86 | 1.8 |
| Spinach | 5.27 | 4.27 | 5.12 | 4.63 | 4.3 |
| Swiss chard | 2.87 | 1.87 | 1.79 | 1.78 | 1.78 |
| Purslane | 4.05 | 4.08 | 3.98 | 4.19 | 4.08 |

Figure S10. Flavonoid contents of vegetables in raw and cooked forms

TAPC (µmol GAE/g DW)

|  | Raw | Steaming | Microwave cooking | Low-water boiling | High-water boiling |
| --- | --- | --- | --- | --- | --- |
| Sorrel | 1.87 | 1.65 | 1.5 | 1.61 | 1 |
| Nettle | 0.19 | 0.1 | 0.02 | 0.03 | 0.02 |
| Spinach | 0.53 | 0.57 | 0.43 | 0.51 | 0.19 |
| Swiss chard | 0.86 | 0.45 | 0.64 | 0.56 | 0.56 |
| Purslane | 0.31 | 0.51 | 0.53 | 0.6 | 0.59 |

Figure S11. Tannin-associated phenolic compounds (TAPC) of vegetables in raw and cooked forms.

DPPH (µmol Trolox/g DW)

|  | Raw | Steaming | Microwave cooking | Low-water boiling | High-water boiling |
| --- | --- | --- | --- | --- | --- |
| Sorrel | 58.47 | 45.11 | 42.88 | 39.26 | 24.44 |
| Nettle | 1.55 | 1.12 | 1.01 | 1.19 | 1.35 |
| Spinach | 5.06 | 3.86 | 4.72 | 4.87 | 3.63 |
| Swiss chard | 4.12 | 2.93 | 2.67 | 4.22 | 3.13 |
| Purslane | 31.56 | 50.54 | 36.14 | 57.12 | 43.87 |

Figure S12. Antioxidant activity levels of vegetables determined by the DPPH method according to cooking methods

ABTS (µmol Trolox/g DW)

|  | Raw | Steaming | Microwave cooking | Low-water boiling | High-water boiling |
| --- | --- | --- | --- | --- | --- |
| Sorrel | 55.79 | 33.89 | 29.48 | 34.45 | 18.03 |
| Nettle | 5.03 | 4.12 | 1.96 | 1.8 | 2.18 |
| Spinach | 10.72 | 6.07 | 8.91 | 7.86 | 5.66 |
| Swiss chard | 7.37 | 2.38 | 2.37 | 3.05 | 1.69 |
| Purslane | 32.45 | 30.91 | 26.17 | 24.02 | 17.49 |

Figure S13. Antioxidant activity levels of vegetables determined by the ABTS method according to cooking methods

CUPRAC (µmol Trolox/g DW)

|  | Raw | Steaming | Microwave cooking | Low-water boiling | High-water boiling |
| --- | --- | --- | --- | --- | --- |
| Sorrel | 106.61 | 86.18 | 71.15 | 59.95 | 43.48 |
| Nettle | 1.21 | 0.93 | 0.46 | 0.58 | 0.59 |
| Spinach | 10.84 | 6.02 | 8.3 | 7.06 | 4.89 |
| Swiss chard | 9.13 | 4.06 | 3.92 | 5.53 | 4.53 |
| Purslane | 62.44 | 62.94 | 35.34 | 79 | 42.32 |

Figure S14. Antioxidant activity levels of vegetables determined by the CUPRAC method according to cooking methods

Figure S15. Percentage change in total phenolic content according to cooking methods.

Rate of change in total phenolic content

|  | Sorrel | Nettle | Spinach | Swiss chard | Purslane |
| --- | --- | --- | --- | --- | --- |
| Steaming | -48.1% | -14.4% | -31.7% | -27.3% | 9.6% |
| Microwave cooking | -56.2% | -41.9% | -25.1% | -33.7% | -7.8% |
| Low-water boiling | -61.6% | -65.2% | -13.8% | -29.3% | 47.8% |
| High-water boiling | -74.1% | -38.5% | -46.4% | -30% | -0.7% |

Rate of change in total phenolic acid content

|  | Sorrel | Nettle | Spinach | Swiss chard | Purslane |
| --- | --- | --- | --- | --- | --- |
| Steaming | 43.5% | 153.2% | -21.9% | -18.6% | 159.5% |
| Microwave cooking | 1.4% | 119.8% | -21.1% | -4.8% | 76.5% |
| Low-water boiling | -1.2% | 279.3% | 14.6% | -7.4% | 275% |
| High-water boiling | -34.7% | 63.1% | 45.7% | -39% | 129% |

Figure S16. Percentage change in phenolic acid content according to cooking methods.

Rate of change in flavonoid content

|  | Sorrel | Nettle | Spinach | Swiss chard | Purslane |
| --- | --- | --- | --- | --- | --- |
| Steaming | -3.07% | -2.44% | -18.98% | -34.84% | 0.74% |
| Microwave cooking | -7.17% | -1.46% | -2.85% | -37.63% | -1.73% |
| Low-water boiling | -4.1% | -9.27% | -12.14% | -37.98% | 3.46% |
| High-water boiling | -5.33% | -12.2% | -18.41% | -37.98% | 0.74% |

Figure S17. Percentage change in flavonoid content according to cooking methods

Rate of change in TAPC

|  | Sorrel | Nettle | Spinach | Swiss chard | Purslane |
| --- | --- | --- | --- | --- | --- |
| Steaming | -11.76% | -47.37% | 7.55% | -47.67% | 64.52% |
| Microwave cooking | -19.79% | -89.47% | -18.87% | -25.58% | 70.97% |
| Low-water boiling | -13.9% | -84.21% | -3.77% | -34.88% | 93.55% |
| High-water boiling | -46.52% | -89.47% | -64.15% | -34.88% | 90.32% |

Figure S18. Percentage change in tannin-associated phenolic compounds (TAPC) according to cooking methods.

Rate of change in DPPH radical scavenging capacity

|  | Sorrel | Nettle | Spinach | Swiss chard | Purslane |
| --- | --- | --- | --- | --- | --- |
| Steaming | -22.85% | -27.74% | -23.72% | -28.88% | 60.14% |
| Microwave cooking | -26.66% | -34.84% | -6.72% | -35.19% | 14.51% |
| Low-water boiling | -32.85% | -23.23% | -3.75% | 2.43% | 80.99% |
| High-water boiling | -58.2% | -12.9% | -28.26% | -24.03% | 39.01% |

Figure S19. Percentage change in DPPH radical scavenging capacity according to cooking methods

Rate of change in ABTS radical scavenging capacity

|  | Sorrel | Nettle | Spinach | Swiss chard | Purslane |
| --- | --- | --- | --- | --- | --- |
| Steaming | -39.27% | -18.09% | -43.38% | -67.71% | -4.75% |
| Microwave cooking | -47.15% | -61.04% | -16.84% | -67.84% | -19.36% |
| Low-water boiling | -38.24% | -64.21% | -26.65% | -58.61% | -25.97% |
| High-water boiling | -67.67% | -56.67% | -47.23% | -77.07% | -46.12% |

Figure S20. Percentage change in ABTS radical scavenging capacity according to cooking methods

Rate of change in CUPRAC values

|  | Sorrel | Nettle | Spinach | Swiss chard | Purslane |
| --- | --- | --- | --- | --- | --- |
| Steaming | -19.16% | -23.14% | -44.46% | -55.53% | 0.8% |
| Microwave cooking | -33.26% | -61.98% | -23.43% | -57.06% | -43.4% |
| Low-water boiling | -43.77% | -52.07% | -34.87% | -39.43% | 26.52% |
| High-water boiling | -59.22% | -51.24% | -54.89% | -50.38% | -32.22% |

Figure S21. Percentage change in CUPRAC values according to cooking methods
